# The role of the endoscope in otologic surgery^☆^

**DOI:** 10.1016/j.bjorl.2019.06.003

**Published:** 2019-06-30

**Authors:** Paulo Valente João

**Affiliations:** Hospital da Pontifícia Universidade Católica de Campinas (PUC-Campinas), Departamento de Otorrinolaringologia, Campinas, SP, Brazil

In recent years, interest in the use of the endoscope during otologic surgery has increased worldwide, and has prompted question including “Is the endoscope better than the microscope?” and “Will the endoscope become the main and predominant instrument from now like in sinus”. These and other questions will probably be better answered in the future, but a reflection based on present and past evidence may perhaps point towards a path or a trend. Thus, the adequate understanding of the instrument (endoscope) and its characteristics, the correct way of using it and of the results are essential.

Regarding the characteristics of the endoscope, it is important to highlight its main advantages and disadvantages. Undoubtedly, the major benefit of the endoscope is to provide better angular vision and better definition, especially due to the close-up capacity. The observation of the middle-ear recesses (retro-tympanum, for instance) is undoubtedly easier and clearer with the use of the endoscope. Another advantage is the possibility of overcoming certain anatomical obstacles that hinder the visualization through a microscope. One example is the external auditory canal, which, depending on its anatomy, creates difficulties for a microscope-assisted transcanal surgical approach (Fig. 1). However, there are some disadvantages. Knowledge of ways to minimize or overcome them is important:^1^

- Performing the surgery with only one hand: since one hand will normally be occupied holding and handling the endoscope, all surgical steps will be performed single-handedly. continuous practicing and adequate hemostasis are very important to minimize this difficulty;
- Loss of three-dimensionality: currently the endoscopes used in otologic surgery do not have three-dimensional (3D) properties. Thus, the lack of binocular vision of the microscope causes a theoretical loss of the sensation of depth. However, the constant movement of the endoscope allows our brain to have this 3D perception, functioning as a “pseudo-three-dimensionality”.
- Possibility of thermal damage: some reports in the literature have suggested that the use of endoscopes can lead to increased temperature in the inner ear.^2^ Actions such as the use of LED light sources and submaximal intensity, recurrent aspiration of the external auditory meatus (cooling of the system) and use of adequate light cables can minimize or virtually exclude the risk of thermal damage to the middle and inner ear structures.

**Figure 1:**
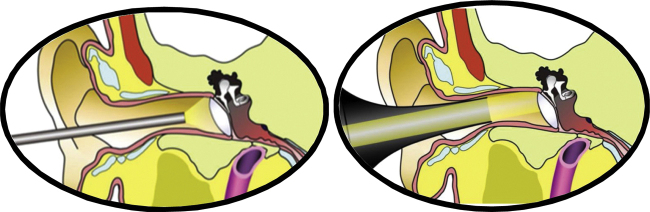
Demonstration of the angular vision characteristic of the endoscope and the capacity to overcome anatomical obstacles, such as the external auditory meatus, compared to the transcanal surgical access using a speculum and microscope.

Another aspect to be remembered is associated with surgical instrumentation. Overall, the instruments are the same as those used in the conventional microscope-assisted technique. However, over the last few years, certain materials have been designed and introduced allowing the refinement of the endoscope-assisted technique. Among them, we can highlight the angulated dissectors and suction tubes. Regarding the endoscope itself three main characteristics have to be discussed: length, diameter and angulation. Most authors recommend the use of longer endoscopes (14 or 18 cm in length), with 3 or 4 mm in diameter (the former may be more suitable in cases of narrow external auditory canal). Regarding the angulation, it can be stated that most procedures and surgical steps can safely be performed with zero-degree optics. However, in certain situations such as the visualization of the middle ear recesses, angulated endoscopes may be important (30 or 45 degrees). Additionally, the use of video systems (cameras and monitors) with high-definition images allow safer surgery, by providing a more adequate and clear visualization of the anatomical structures.

In the context of otologic surgery, the endoscope can be used from a tympanotomy for the placement of a ventilation tube, to the resection of vestibular schwannomas. In general, the endoscope can be useful by allowing an innovative, less invasive approach or simply to have more adequate performance of a certain surgical step. This is evident, for instance, in the treatment of chronic otitis media. In tympanoplasty, the endoscope facilitates the transcanal access.^3^ In the cholesteatoma surgery, in turn, it provides better visualization of the middle ear recesses, such as those of the retro-tympanum (Fig. 2).

**Figure 2:**
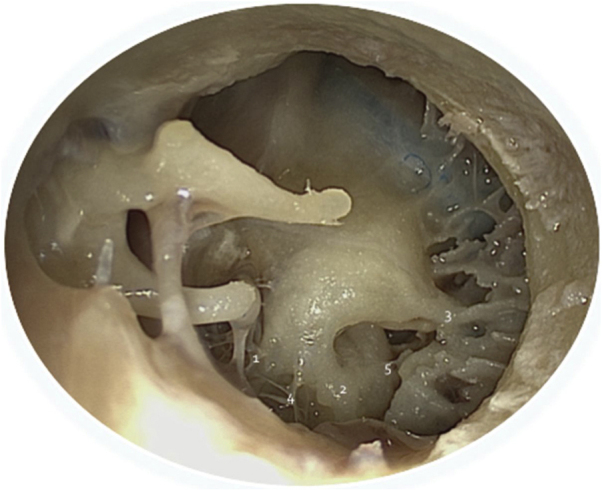
Anatomy of the retro-tympanum (endoscopic view); 1- Ponticulus; Subiculum; 3- Finiculus, 4 - Sinus tympani, 5 - Sinus subtympanicus.

In order to establish the endoscope as a useful instrument for otologic surgery, it is important to analyze and publish its outcomes. In this direction, the literature accuracy has gradually increased. Ayache in 2013 published the results of 30 patients submitted to tympanoplasty with cartilage graft through endoscope-assisted transcanal approach. The success rate (closure of the perforation one year after the procedure) was 96%, comparable to techniques using the microscope.^3^ More recently, Tseng studied the results of 91 patients submitted to tympanoplasty. In this study, 87.9% of the patients had perforation closure 3 months after surgery and 86.8% had air-bone gap closure in audiometry (<20 dB).^4^ In 2016, Marchioni and colleagues presented the results of 234 patients with cholesteatoma submitted to endoscopic surgery (with or without mastoidectomy). The recurrence rate and residual disease after 64.3 months (mean follow-up time) was 68%, comparable to that of traditional techniques.^5^ In the context of stapedectomies, a recent meta-analysis suggested that the audiological results of the endoscopic technique would be similar to those of the microscope-assisted technique, with a lower incidence of the necessity of curettage of the scutum, and manipulation of the chorda tympani nerve and consequent dysgesia in the postoperative period.^6^

Another relevant aspect that is worth mentioning is the learning curve. As in any area of surgical Medicine, the adaptation or the adoption of a certain technique takes a certain time. Therefore, benefits and outcomes will be worse at the beginning. Pothier recently wrote that the learning curve of endoscopic-assisted otologic surgery may be longer and more difficult, especially for surgeons not accustomed to using the endoscope and, therefore, he suggests that the instrument introduction be made slowly and progressively.^7^

Based on the above mentioned facts, currently it cannot be stated today that the endoscope is or will be better than the microscope in the context of otologic surgery. And, in fact, this discussion may not be relevant, since they are distinct instruments that have advantages and disadvantages for a surgical procedure or an specific step. Perhaps the most important is the fact that the surgeon should have adequate knowledge of both instruments, their characteristics and specificities, in order to apply them correctly, aiming always a better result for patients. Much more than antagonistic, the endoscope and the microscope are simply complementary instruments.

## Conflicts of interest

The authors declare no conflicts of interest.
